# Conserved Genes Underlie Phenotypic Plasticity in an Incipiently Social Bee

**DOI:** 10.1093/gbe/evy212

**Published:** 2018-09-22

**Authors:** Sandra M Rehan, Karl M Glastad, Michael A Steffen, Cameron R Fay, Brendan G Hunt, Amy L Toth

**Affiliations:** 1 Department of Biological Sciences, University of New Hampshire; 2 Department of Cell & Developmental Biology, University of Pennsylvania; 3 Department of Ecology, Evolution and Organismal Biology, Iowa State University; 4 Department of Entomology, University of Georgia

**Keywords:** social transitions, phenotypic plasticity, molecular evolution, comparative genomics, taxonomically restricted genes, small carpenter bee

## Abstract

Despite a strong history of theoretical work on the mechanisms of social evolution, relatively little is known of the molecular genetic changes that accompany transitions from solitary to eusocial forms. Here, we provide the first genome of an incipiently social bee that shows both solitary and social colony organization in sympatry, the Australian carpenter bee *Ceratina australensis*. Through comparative analysis, we provide support for the role of conserved genes and cis-regulation of gene expression in the phenotypic plasticity observed in nest-sharing, a rudimentary form of sociality. Additionally, we find that these conserved genes are associated with caste differences in advanced eusocial species, suggesting these types of mechanisms could pave the molecular pathway from solitary to eusocial living. Genes associated with social nesting in this species show signatures of being deeply conserved, in contrast to previous studies in other bees showing novel and faster-evolving genes are associated with derived sociality. Our data provide support for the idea that the earliest social transitions are driven by changes in gene regulation of deeply conserved genes.

## Introduction

Considered one of the major evolutionary transitions of life on earth, the evolution of eusociality, typified by overlapping generations, cooperative brood care, and reproductive division of labor, has been of great interest to biologists for over a century (Szathmáry and Maynard Smith 1995). This major transition has accompanied dramatic increases in functional complexity, ecological role, and niche breadth in eusocial lineages (Wilson 1971; Michener 1974; Hölldobler and Wilson 1990). However, despite a strong history of theoretical work into the mechanisms of social evolution, relatively little is known of the molecular genetic changes that accompany transitions from solitary to eusocial forms (Robinson 1999; Bloch and Grozinger 2011; Kapheim et al. 2015; Patalano et al. 2015). Transitions from solitary to incipient societies have been predicted to involve changes in timing or location of gene expression (Rehan and Toth 2015). In effect, all individuals of incipient insect societies remain capable of performing all tasks, including foraging and reproduction, with distinct roles mediated by environmental pressures and regulatory plasticity of pre-existing genes (West-Eberhard 2003). As increasingly more complex social interactions evolve, where single foundress nests transition into cooperative colonies with the emergence of worker daughters, social roles can become fixed with more permanent and distinct gene expression patterns. Further along the social spectrum, distinct castes, and division of labor becomes the hallmark of primitive and advanced eusocial societies, and are associated with large differences of gene expression between castes (Grozinger et al. 2007; Ometto et al. 2011). It is predicted that genes predominantly only needing to function in an individual caste are released from pleiotropic constraints allowing selection for changes in gene sequence that may facilitate the elaboration of derived social traits (Gadagkar 1997). Additionally, because individuals within advanced eusocial insect colonies only need to perform a subset of tasks, gene duplication, followed by genetic release and diversifying selection can be particularly strong in producing elaborate traits (Gadagkar 1997; Chau and Goodisman 2017). Positive selection on genes related to social traits, as well as an increased role for “novel,” taxonomically restricted genes are predicted to become increasingly prevalent during the evolution of highly eusocial behavior (Rehan and Toth 2015).

In the context of this conceptual framework, as lineages climb the “social ladder” to more complex sociality, evolutionary changes in gene expression and regulatory evolution are predicted to be essential in the incipient social transitions (West-Eberhard 1987, 1996). Therefore, understanding of the genetic mechanisms of increasing levels of social complexity must include taxa that may represent the incipient stages in the evolution of eusociality (Rehan and Toth 2015; Rehan et al. 2016; Toth and Rehan 2017). However, despite the importance of the inclusion of the many social forms in sociogenomic analysis, studies still primarily focus on eusocial species, with data on species displaying simpler social structure largely lacking.

**Fig. 1.**
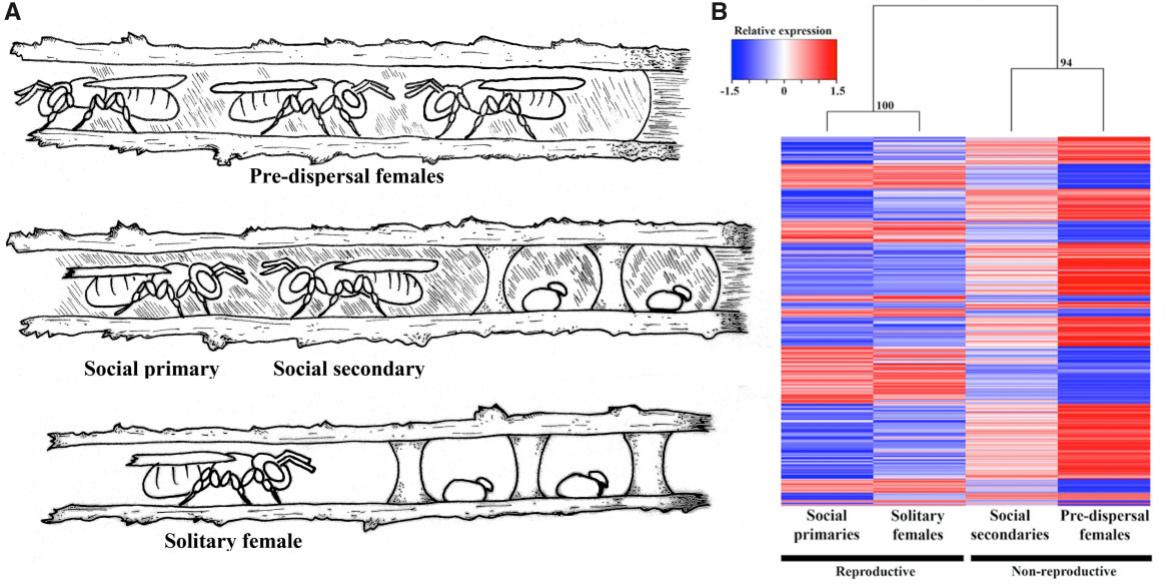
—(*A*) Nesting biology of *C. australensis.* Top: predispersal nest containing multiple females (callow, predispersal females). Middle: social nest with a social primary and social secondary female (social nests formed when sisters remain at the natal nest to cooperatively reproduce and darkened walls indicate nest reuse). Bottom: nest with a single, solitary nesting female (solitary females disperse and establish a new nest as shown with clean nest walls). (*B*) Heatmap of all significantly differentially expressed genes (FDR corrected *P* values < 0.05; n = 1591) by behavior class, with three biological replicates of three individual brains per class. Blue = downregulated, red = upregulated, white = not differentially expressed among classes, relative expression values = log_2_(fold change). Hierarchical clustering analysis shows high support for two major categories, reproductive versus nonreproductive. Social primaries and solitary females comprise the reproductive category (bootstrap support 100 PP), and social secondaries and predispersal females form a strongly supported nonreproductive clade (94 PP).

The small carpenter bees (genus: *Ceratina*) are an excellent group to test hypotheses regarding the evolution of incipient sociality. In *Ceratina*, most species are solitary, in as much as only a single female attends to her offspring, but sociality is known to occur in some species. However, sociality in *Ceratina* is never observed to the extent seen in the advanced eusocial bee species (Michener 2007). The genus is highly diverse and is widely distributed across all continents (excluding Antarctica) with a single species in Australia, *Ceratinaaustralensis* (Michener 2007). *Ceratinaaustralensis* is of special interest to the study of social evolution because it is an incipiently social and socially polymorphic species with both solitary and social nests occurring in the same population and at the same time of year (fig. 1*A*; Rehan et al. 2010, 2011, 2014). In solitary nests, females forage and reproduce independently. In social nests, the primary female behaves much like a solitary female, monopolizing foraging and reproductive duties, whereas a secondary (sibling) female remains at the nest as a guard (Rehan et al. 2010). Females make their nests in the pith of dead, broken twigs. Solitary nests are formed when a single female disperses to find and establish a new nest and social nests are formed from two sisters remaining at the natal nest (Rehan et al. 2011). This social polymorphism within populations provides a natural experiment to explore the molecular changes that may underlie the transition from solitary to social life within a single species (Rehan et al. 2010, 2011, 2014). The nest-sharing behavior of *C. australensis* represents one of most fundamental types of social behavior found in bees, and this type of incipiently social cooperation and rudimentary division of labor at nest founding may have paved the way for subsequent transitions to caste-containing societies.

Here, we present new genome and transcriptome data for the Australian small carpenter bee, *C. australensis*; this represents the first study comparing genomic and transcriptomic data for an incipiently social species. We compare these data to the previously published bee genomes to identify distinct genomic features of this bee compared with previously sequenced bee genomes, including gene family expansions and genes with signatures of positive selection. Additionally, we identify transcriptomic differences between socially polymorphic individuals within the same population by investigating four different reproductive and foraging physiologies: social primaries (reproductive and foraging), social secondaries (nonreproductive and nonforaging), solitary active brood females (reproductive and foraging), and predispersal females that are newly eclosed (prereproductive and preforaging). We further expand these analyses with a systems level approach by characterizing transcription factors conserved across independent origins of sociality using other existing genomic and transcriptomic data.

Using these data, we fill in knowledge gaps about incipient social evolution by addressing three questions and testing explicit predictions on the molecular evolution of incipient sociality (Rehan and Toth 2015); we argue that sociality is predicted to have evolved from ancestral behavioral and physiological phenotypic plasticity, so we might expect an emphasis on gene regulation rather than protein evolution. First, what role do taxonomically restricted or “novel” genes have in incipient sociality relative to conserved genes? We predict that relatively conserved and ancient, rather than more recent novel genes should be involved in incipiently social relative to eusocial phenotypes (Rehan and Toth 2015; Toth and Rehan 2017). Second, is there evidence of positive selection and evolutionary changes in gene regulation in the incipient evolution of sociality? We predict that evolutionary changes occurring at the DNA sequence level in incipiently social species should be related to gene regulation, such as protein coding changes in transcription factors and changes in the sequence of transcription factor binding sites (Rehan and Toth 2015). Third, is there evidence that conserved genes have been functionally coopted during social evolution? A general hypothesis of evo-devo and social evolution is that a shared ancestral genetic toolkit should be conserved across social lineages (Rehan and Toth 2015; Toth and Rehan 2017). Accordingly, we predict that genes associated with incipient sociality in *C. australensis* will also be associated with caste differences in advanced eusocial species (Toth and Robinson 2010).

## Materials and Methods

### Sample Collection and Preparation

Adult female bees were collected at dawn and dusk from individual active nests in Warwick, Queensland, Australia in December 2014. Upon nest dissection bees were flash frozen in liquid nitrogen for subsequent brain dissection and RNA extraction as well as ovarian dissection and wing wear scoring. Bees were separated into four behavioral categories and classified as follows: social primaries (reproductive and foraging, with visible wing wear and one of two bees in a social, active brood rearing nest), social secondaries (nonreproductive and nonforaging, with no wing wear and the second of two bees in a social, active brood rearing nest), solitary females (reproductive and foraging, lone females in with actively developing brood), and predispersal females (nonreproductive and nonforaging, newly eclosed females from solitary nests). Brood rearing seasons in this species are bivoltine and largely synchronous (Rehan et al. 2010, 2011, 2014). As such, solitary, social primary, and secondary females are all dark winged, with dense integument, and from the summer brood cohort (∼10 months old). The predispersal females are all light-winged, with soft integument/newly eclosed and from the spring brood (<1 week old). Behavioral categories are discrete with marked differences in wing wear and ovarian development allowing for clear classification of females in the nest (Rehan et al. 2010, 2011, 2014).

We used the RNeasy Mini Kit (Qiagen) to extract total RNA from brain tissue of nine females for each of four behavioral categories, three pooled brains per replicate and three replicates per behavioral category. Brain tissue was used due to its relevance to behavior and comparative studies (Grozinger et al. 2007; Ferreira et al. 2013; Rehan et al. 2016). RNA quality was assessed using spectrophotometry (NanoDrop) and an Agilent BioAnalyzer. RNAseq libraries were prepped using TruSeq RNAseq Sample Prep kit with 250 ng of RNA, which included Poly(A) RNA purification, fragmenting using sonification, cDNA synthesis from sized selected fragments (∼260 nucleotides) using random primers, and barcoding.

Using two lanes on an Illumina HiSeq 2500 sequencing machine, we generated an average of 18.5 million 150 base paired-end reads for all samples. Raw data have been submitted to the NCBI Sequence Read Archive (SRA) with accession number PRJNA302037. FastQC was used to visualize raw reads from each library to determine data quality. Adapter sequences were removed and reads were filtered for quality (threshold ≥20 and length threshold of 50 bases). This process removed ∼20% of the reads. Transcript abundance for each library was quantified using HTseq (Version 0.6.2) from alignments of the raw paired-end reads to the *C. australensis* genome made using Bowtie2 (Version 2.1.0).

Details of genome sequencing, assembly, annotation, gene expression, transcription factor enrichment, molecular evolution, and phylostrata analyses are given in supplementary methods, Supplementary Material online.

## Results and Discussion

### Genome Composition of the Australian Small Carpenter Bee

The estimated genome size of *C. australensis* is well within the typical range of other bees, at ∼233 Mb and the final assembly has an N50 of 168 kb and a total length of 219.3 Mb (supplementary tables S1 and S2, Supplementary Material online). The assembly appears to cover much of the gene space of this species; of 248 core eukaryotic genes, 247 were completely assembled in the *C. australensis* genome and analysis of Benchmarking Universal Single–Copy Orthologs (BUSCO) genes showed that the assembly contains 87.7% complete arthropod BUSCO orthologs (supplementary table S2, Supplementary Material online). A combination of RNA-sequencing, de novo, and homology-based predictions generated the official gene set of 16,386 predicted genes. These 16,386 predicted genes comprise 7,264 gene families (supplementary fig. S1, Supplementary Material online). A total of 7,070 gene families are shared among all bee species used in our comparison. Within *C. australensis* there are 67 predicted unique gene families in relation to all other bee genomes (supplementary fig. S1 and table S3, Supplementary Material online); among which include zinc finger gene families with GO enrichment for transcription factor activity. Gene family expansions are of interest because they have the potential to provide insight into molecular functional processes under selection. Within the subfamily Xylocopinae, which comprises both *C. australensis* and *Ceratinacalcarata*, OrthoMCL (Li et al. 2003) identified 161 expanded gene families in comparison with all other bee lineages (supplementary table S4 and figs. S1 and S2, Supplementary Material online). Of these gene families, there are expansions of numerous transcription factors (12 gene families), including zinc finger proteins. *Ceratinaaustralensis* shows a large expansion of zinc finger proteins, possessing 10 more of these genes than the next closest subsocial relative, *C. calcarata*, a congener without cooperative brood care (supplementary table S4, Supplementary Material online). This is of interest because some members of this gene family have been implicated in the regulation of female reproduction (Terrapon et al. 2014). Additionally, the number of binding sites for zinc finger proteins are suggested to have been expanded in eusocial bee species relative to solitary species (Kapheim et al. 2015).

There are also noteworthy expansions of several metabolic gene families in *C. australensis*. Of interest is the expansion of fatty acid desaturase genes (supplementary table S4, Supplementary Material online), known to have important roles in chemical communication and to be especially diversified in ants (Hazel and Williams 1990; Helmkampf et al. 2015). We also find expansion of the insect pheromone-binding gene family, a family associated with chemical communication (Pelosi et al. 1995). Another notable expansion includes the stathmin gene family (supplementary table S4, Supplementary Material online), which is implicated in fear response, parental care, and adult social behavior in mice (Martel et al. 2008).

### Zinc Finger Transcription Factors have Elevated Rates of Protein Sequence Change in *C. australensis*

Evolutionary developmental, as well as social theory, hypothesize that novel traits largely evolve by changing the timing and/or expression of functionally conserved genes, and that such changes can largely occur through cis-regulatory evolution (Carroll 2008; Rehan and Toth 2015). Here, we treat changes in the protein coding sequences of transcription factors as a special case considering evo-devo theory. We predict downstream gene expression changes largely occur through both cis-regulatory evolution and selection on coding sequences of key transcription factors. Thus, we would predict accompanying protein expression changes largely occur through cis-regulatory evolution and selection on key transcription factors. For *C. australensis*, PAML analysis comparing rates of nonsynonymous (d*N*) to synonymous (d*S*) nucleotide substitutions (see supplementary methods, Supplementary Material online) found 153 genes that displayed a significantly faster rate of sequence evolution in *C. australensis* than in the background of all other bee lineages (supplementary table S5, Supplementary Material online), including six different zinc finger proteins. For example, there is strong evidence for positive selection for zinc finger 846-like protein (d*N*/d*S* = 5.8), a gene implicated in DNA binding from human studies (Rolland et al. 2014). Taken together, these 153 genes had significant GO enrichment for postsynaptic specialization, nucleotide binding, and protein metabolic processes (supplementary table S6, Supplementary Material online).

### Numerous Genes Show Brain Expression Patterns Related to Incipient Sociality

The social ladder hypothesis predicts that changes in gene regulation are likely to predominate in the earliest social transitions (Rehan and Toth 2015). As a starting place to identify genes associated with incipient sociality, we used RNA-sequencing of brain tissue to characterize patterns of differential expression between bees exhibiting different behavioral states (fig. 1). Using DESeq (Anders and Huber 2010), we identified 1,591 total DEGs (differentially expressed genes) across the four behavioral groups (supplementary table S7, Supplementary Material online). Of these DEGs, 836 have significant homology to known proteins using Blast2GO (Conesa et al. 2005), 83 are uncharacterized proteins, and the other 672 have no known homology (supplementary table S7, Supplementary Material online).

Comparing the nest-sharing females, we find 59 DEGs between social primaries and secondaries, and neurobiological GO terms associated with regulation and secretions of neurotransmitters as well as pheromone production, and light stimulus and activity were enriched in social primaries over social secondaries (FDR ≤ 0.05; supplementary tables S7 and S8, Supplementary Material online). Comparison of brain gene expression in age matched solitary versus social (primary and secondary) females revealed 382 DEGs (supplementary table S9, Supplementary Material online). Genes upregulated in social females include odorant-binding proteins 1 and a10 which are important for chemical communication in insects (Pelosi et al. 1995). Metabolic process GO terms associated with carbohydrate and protein metabolism were enriched in social over solitary females (Kapheim et al. 2015) as well as RNA modification and translation biological processes (supplementary table S10, Supplementary Material online). Genes that are differentially expressed between conspecific females differing in social behavior have been frequently used in the literature as candidate “sociality” genes for species of interest (Gadagkar 1997; Hunt et al. 2010; Harpur et al. 2014; Berens et al. 2015). Although none of these genes have demonstrated causal roles, the fact that they differ in expression between social forms, and that many are conserved across species, suggests they can be considered as informative candidate genes for the regulation of sociality and its evolution.

**Fig. 2.**
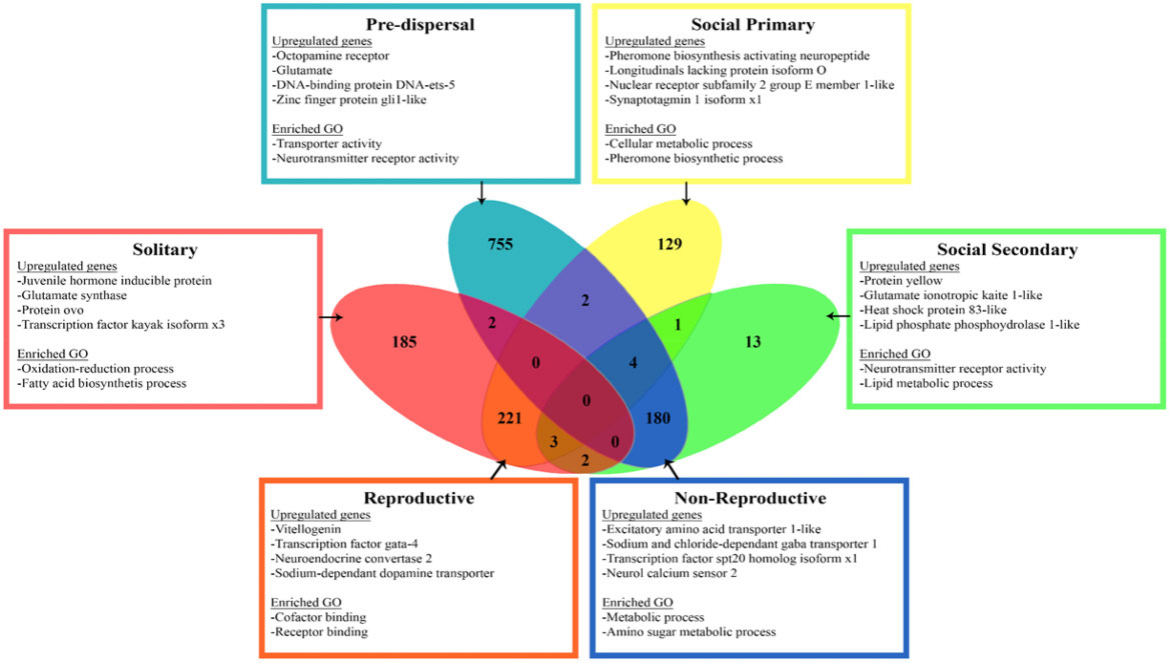
—Venn diagram depicting the relationship of significant upregulation in genes in the four behavioral categories, as well as the subcategories of reproductive (solitary and social primary) and nonreproductive (predispersal and social secondary) groupings. Boxes show specific upregulated genes and enriched GO terms for each category.

Examining reproductive (solitary and social primary) and nonreproductive (predispersal and social secondary) females show large differences in gene expression, with 934 DEGs. Hierarchical clustering indicates that the two reproductive female categories showed the highest gene expression similarity, with only 11 genes differentially expressed between solitary and social primary females (fig. 1*B*). The largest number of DEGs are found between the predispersal females and reproductive individuals (primary and solitary females), with 925 and 1,215 DEGs respectively. DEGs present include cuticular and chitin formation proteins, *glucose* and *sorbital dehydrogenase*, and *aldose reductase*, all of which are important in physiology, metabolism, and development (Wolfe et al. 1998; Petrash 2004; Tang et al. 2015). Included in the DEGs are transcription factors, such as the *transcription factor castor* (supplementary table S7, Supplementary Material online), which is central in the developing central nervous system (Mellerick et al. 1992). Pheromone/odorant genes are also differentially expressed between predispersal and reproductive females, including two different odorant receptors (fig. 2). Interestingly, nonreproductive categories (predispersal and social secondary females) have relatively few (25) DEGs between them. This is likely attributable to the fact that both nonreproductive categories are also nondispersing and nonforaging females, known to have reduced mushroom body development in comparison with solitary and social primary females (Rehan et al. 2015).

### Genes Related to Incipient Sociality Are Associated with Regulatory Regions with Neural and Behavioral Functions

The whole genome sequence of *C. australensis* allows us for the first time to examine noncoding sequence of an incipiently social bee and identify transcription factor (TF) binding motifs related to this rudimentary form of sociality. Using the MEME program suite (Bailey et al. 2009) to identify motifs in our focal genome, 63 transcription factor (TF) binding motifs are enriched (compared with nondifferentially expressed gene sequences) across all differentially expressed *C. australensis* genes (*N* = 1,591; supplementary table S11, Supplementary Material online). Many of these motifs are binding sites of genes important in neural development and differentiation (supplementary table S11, Supplementary Material online). Examples include *Pox meso*, a transcription factor important in dendrite morphogenesis (Iyer et al. 2013), and *Adh transcription factor 1*, a transcription factor that regulates genes important in memory and olfactory learning in *Drosophila* (DeZazzo et al. 2000). We also identified additional transcription factor binding motifs, such as for *Hairy* (associated with genes upregulated in solitary over predispersal females) which has a known function in neuron fate and axonogenesis (Demidenko et al. 2001; Grueber et al. 2007; Monastirioti et al. 2010; table 1 and supplementary table S11, Supplementary Material online). Additionally, the motif *gooseberry* (associated with genes upregulated in social primaries and solitary reproductives over nonreproductive social secondary and predispersal females) transcription factor is enriched (supplementary table S11, Supplementary Material online). This transcription factor is known to be important in neural development (Demidenko et al. 2001; Grueber et al. 2007; Neumüller et al. 2011).

**Table 1:** A Selection of Matches to 13 Transcription Factor Binding Motifs Associated with Significantly DEGs (FDR *P* < 0.05)

Motif	Function	Species
Adf1	Memory, synapse assembly	*A. mellifera*
cwo	Dendrite morphogenesis	*Drosophila melanogaster*
Egr1	Neuroplasticity	*M. musculus*
gsb	Neurogenesis, regulation of synaptic activity	*Drosophila melanogaster*
klu	Neurogenesis	*Drosophila melanogaster*
Med	Neuron development, synaptic growth	*Drosophila melanogaster*
Met	Juvenile hormone binding	*A. mellifera*
ovo	Adult feeding behavior, pheromone metabolic process	*Drosophila melanogaster, M. musculus*
Poxm	Dendrite morphogenesis	*Drosophila melanogaster, Danio rerio*
pros	Axonogenesis, brain development	*Drosophila melanogaster*
Sr	Central nervous system development	*Drosophila melanogaster*
Tgo	Brain development	*Drosophila melanogaster*
CREB2	Neuroplasticity and long-term memory	*Danio rerio*

Note.—A full list of motifs, matches, and references may be found in supplementary table S9, Supplementary Material online.

### Differentially Expressed Genes in Incipiently Social Bees Are Evolutionarily Ancient

**Fig. 3.**
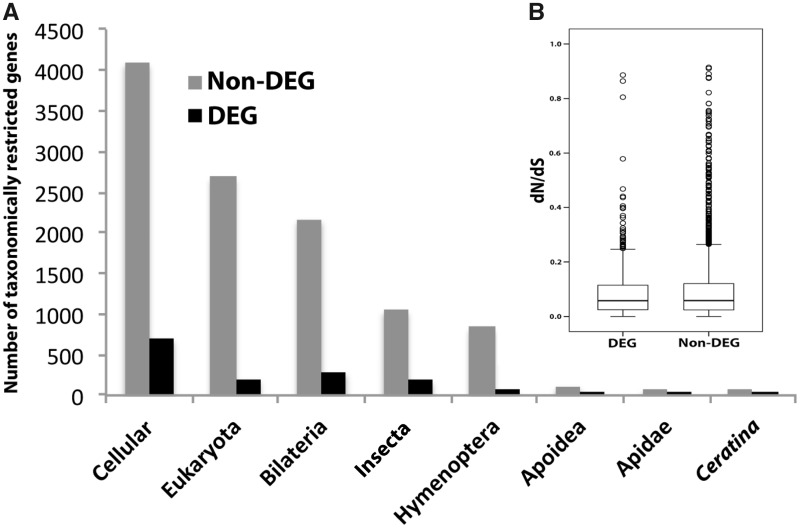
—(*A*) Distribution of differentially expressed genes (DEGs) across eight phylostratigraphic levels. DEGs are overrepresented among ancient conserved genes relative to non-DEGs (cellular to Insecta vs Hymenoptera to *Ceratina*; χ^2^=13.853, df = 1, *P* < 0.001; supplementary tables S7 and S13, Supplementary Material online). See supplementary table S12, Supplementary Material online, for full description of taxonomic designations. (*B*) Comparison of average d*N*/d*S* between genes that were differentially expressed in *C. australensis* females (*N* = 492) versus those that were not differentially expressed (*N* = 2936). DEGs and non-DEGs have similar rates of protein evolution (Mann–Whitney *U*, *Z*=−0.366, *P* = 0.71).

Previous studies have suggested novel genes, or genes that are evolutionarily more recent, are associated with highly eusocial traits (Johnson and Tsutsui 2011; Ferreira et al. 2013). As a corollary, the social ladder hypothesis predicts the earliest social transitions to be associated with deeply conserved and ancient genes (Rehan and Toth 2015). We used phylostratigraphic analysis, which designates individual genes to predetermined taxonomic levels based on evolutionary age, to assess the relative ages of differentially expressed genes observed in an incipiently social species. This analysis assigned 11,065 genes to eight taxonomic levels (fig. 3*A* and supplementary tables S12–S14, Supplementary Material online), with most genes being deeply conserved in all cellular organisms, followed by Eukaryota, Bilateria, Insecta, Hymenoptera, Apoidea, Apidae, and *Ceratina*. Differentially expressed genes are more highly represented than nondifferentially expressed genes in the most ancient phylostrata (Cellular to Insecta; 12% DEGs, 78% non-DEGs) compared with more recent phylostrata (Hymenoptera to *Ceratina*; 1% DEGs, 9% non-DEGs; χ^2^=13.853, df = 1, *P* < 0.001; supplementary tables S13–S15, Supplementary Material online). This overall pattern was consistent across comparisons of reproductives versus nonreproductives (supplementary fig. S3, Supplementary Material online), social primaries versus social secondaries (supplementary fig. S4, Supplementary Material online), and solitary versus social primaries (supplementary fig. S5, Supplementary Material online). Ancient genes consistently represent the clear majority of differentially expressed genes, supporting the idea that evolutionary ancient genes rather than novel genes underlie incipiently social behavioral traits (Rehan and Toth 2015). This is consistent with the idea that ancient genes underlie behaviors under pleotropic constraint such as reproduction and parental care, whereas novel genes are thought to evolve during later stage sociality as seen after genetic release and obligate division of labor (Simola et al. 2013; Feldmeyer et al. 2014; Harpur et al. 2014; Kapheim et al. 2015).

### Genes Associated with Incipient Sociality Are Not Rapidly Evolving

To examine rates of evolution of genes associated with incipient sociality, we examined d*N*/d*S* ratios for the DEGs associated with the four *C. australensis* behavioral states. We found no significant difference in the rate of molecular evolution between overall DEGs and non-DEGs, both with estimated rates of evolution being very low (Mann–Whitney *U*, *Z* = −0.366, *P* = 0.714; fig. 3*B*). Likewise, we found no significant difference in the rate of molecular evolution between solitary and social (primary and secondary) DEGs (Mann–Whitney *U*, *Z* = −1.031, *P* = 0.303; supplementary table S15, Supplementary Material online). This stands in contrast to results from eusocial bees, suggesting elevated rates of sequence evolution of genes associated with eusocial traits (Hunt et al. 2011; Johnson and Tsutsui 2011; Harpur et al. 2014). However, we found that when investigating specific behavioral states, upregulated genes in the reproductive categories show significantly higher d*N*/d*S* compared with non-DEGs (Mann–Whitney *U*, *Z* = −2.493, *P* = 0.013; supplementary table S15, Supplementary Material online). Elevated rates of sequence evolution in reproductive genes are a common phenomenon across most taxa (Swanson and Vacquier 2002; Clark et al. 2006; Hunt et al. 2010). The decoupling of reproduction and foraging genes seen in eusocial taxa allows for the circumstance of derived worker traits showing higher rates of evolution (Gadagkar 1997), but see (Harpur et al. 2017). These data from *C. australensis* suggest DEGs in incipiently social taxa are not under relaxed purifying or positive selection.

### Shared Patterns of Gene Expression across a Social Spectrum

The conserved genomic toolkit hypothesis suggests that regulatory changes in specific genes and pathways, especially those related to core, conserved organismal functions, are central in the evolution of sociality across independently evolved social lineages (Toth and Robinson 2010). Accordingly, we predict that conserved genes should be associated with caste differences in advanced eusocial species, as well as incipiently social species in independently social taxa (Rehan and Toth 2015).

**Fig. 4.**
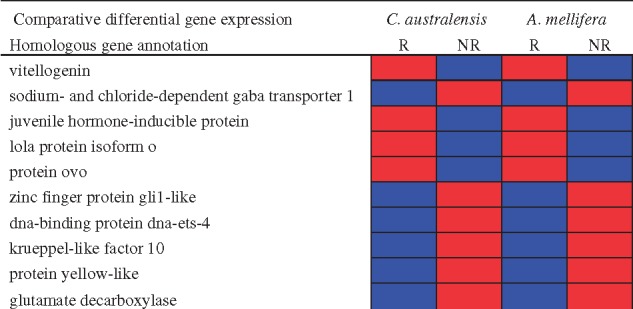
—Conserved genes and regulation patterns between reproductive (R) and nonreproductive (NR) individuals in incipiently social (*C. australensis*) and advanced eusocial (*A. mellifera*) brain gene expression studies. Significantly upregulated genes shown in red and downregulated genes shown in blue. A selection of the top ten highly expressed, behaviorally relevant, and differentially expressed genes is present here. The full list of genes and references may be found in supplementary table S7, Supplementary Material online.

In order to assess whether shared genes are associated with incipient sociality in *C. australensis* and social behavior in other, independently evolved social taxa, we performed comparisons of *C. australensis* DEGs to published findings on social aggression, dominance, and development in both vertebrate and invertebrates, from 17 different taxa (12 insect, 2 mammal, 2 fish, and 1 bird species; supplementary table S16, Supplementary Material online) using hypergeometric tests to detect significant overlapping gene lists. The largest overlap in shared DEGs is found when comparing to studies of caste differentiation in other social Hymenoptera (supplementary table S7, Supplementary Material online) (Grozinger et al. 2007; Rehan et al. 2014). One of the commonly overlapping genes is *vitellogenin* (*Vg*), which is typically upregulated in reproductive females (fig. 4). Additionally, several genes involved in neurobiological function are differentially regulated between castes of *Apismellifera* and between reproductive and nonreproductive individuals in *C. australensis*, including two genes important in the function of the neurotransmitter glutamate (sodium and chloride-dependent GABA, glutamate decarboxylase; fig. 4) (Cardeon et al. 2011; Cameron et al. 2013). DEGs important in the regulation of juvenile hormone, a hormone important in the development and behavioral maturation of insects (Nijhout 1994; Sullivan et al. 2000) also show overlap with studies on honey bee (fig. 4) and paper wasp castes (Cardeon et al. 2011; Ament et al. 2012; Toth et al. 2014).

In order to address overlap of functional gene categories across social species, significantly enriched GO terms in *C. australensis* were compared with 27 different studies, representing 23 different species, for their roles in aggression, social dominance, and development (17 insect, 2 mammal, 3 fish, and 1 bird species; supplementary table S16, Supplementary Material online). The largest overlap in GO term enrichment is found among studies investigating aggressive behavior in bees, ants, and wasps (supplementary table S8, Supplementary Material online) (Alaux et al. 2009; Rittschof et al. 2014) GO terms for signal transduction and synaptic transmission are notably common across studies (supplementary table S8, Supplementary Material online). Genes related to synaptic transmission were differentially expressed across seven studies investigating aggression and colony formation in a broad range of taxa (*A.mellifera, Solenopsis invictus*, *C.calcarata*, and *Mus musculus*; supplementary table S7, Supplementary Material online).

Common transcription factor binding motifs enriched from *C. australensis* DEGs were detected across eight studies from nine taxa (6 insect, 2 fish, 1 mammal; table 1). The motif for *Adh Transcription Factor 1* (*Adf1*), a transcription factor associated with learning and memory is enriched in association with DEGs from our study, and is also enriched in several other studies related to aggression in insects (DeZazzo et al. 2000; Cristino et al. 2006; Withee and Rehan 2017). Taken together, these results indicate *C. australensis* behavior is related to shared genes, pathways, and regulatory elements deeply conserved in association with social behavior across both invertebrate and vertebrate behavioral comparisons (supplementary tables S7 and S16, Supplementary Material online).

## Conclusions

Here, we present the first genomic investigation of incipient sociality in *C. australensis*, a carpenter bee that is part of a lineage with both solitary and highly eusocial members. Genomic analysis of this species allowed us to test aspects of the social ladder hypothesis in the evolution of incipient sociality, a part of the social spectrum largely neglected. First, we asked what role do taxonomically restricted genes have in incipient sociality relative to conserved genes. Our results point to a role for gene regulatory evolution and conserved genes in incipient social evolution. In contrast to previous studies on bees, wasps, and ants with more highly derived sociality, our results show no support for fast-evolving, novel genes to be associated with incipiently social phenotypes. The novel gene hypothesis proposes that eusociality, as a novel phenotype, arose via the evolution of taxonomically restricted genes (Johnson and Tsutsui 2011). Support for this hypothesis generally comes from highly eusocial species where signs of positive selection are found in these novel genes (Simola et al. 2013; Feldmeyer et al. 2014; Harpur et al. 2014; Kapheim et al. 2015). However, in *C. australensis*, we find that there is little evidence for a predominant role of novel genes in incipient social evolution. The clear majority of *C. australensis* genes that are associated with the social polymorphism have evidence for ancient origins, and on an average have a similar rate of protein evolution as other genes in the genome.

This finding is in general agreement with predictions of the social ladder hypothesis, suggesting less involvement of novel genes in incipient social evolution. Instead, the data suggest that conserved genes are more relevant to incipient social evolution. Our second and third questions asked if there is evidence for evolutionary changes in gene regulation in incipient social evolution, and if conserved genes have been coopted and functionally rewired during social evolution. The social ladder hypothesis, grounded in evo-devo considerations states that changes in the regulation of deeply conserved genes, or “genetic toolkits,” are predicted to dominate at the incipient stages of social evolution (Rehan and Toth 2015). This prediction is largely supported in this study based on three observations: 1) genes associated with regulation of expression, for example, zinc finger transcription factors, show evidence of protein sequence evolution as well as gene family expansions in an incipient stage social taxon, 2) many genes differentially expressed in association with *C. australensis* social plasticity are deeply conserved genes (fig. 3*A*), and 3) conserved patterns of differential gene expression and associated transcription factors are linked to social plasticity in both *C. australensis* and advanced social insects (supplementary tables S7 and S16, Supplementary Material online). While there is some evidence that ancient genes may have more cis-regulatory evolution in *Drosophila* (Wittkopp et al. 2004; reviewed in Simpson 2007), this is a new line of research worth further investigation among social insect taxa.

Social insect genomes provide unparalleled insights into the genetic basis of phenotypic plasticity and social organization (Patalano et al. 2015). Our analysis of the genomic mechanisms underlying social structure in *C. australensis* shows common, deeply conserved genetic mechanisms of sociality compared with other bees and other social taxa. By providing genomic resources in a phylogenetic context, our study fills a critical gap in our knowledge of the genomic basis of social transitions in the evolution of eusociality. Our findings indicate relatively low rates of protein sequence change, and few novel genes associated with the earliest social transitions. Instead, our results highlight evolutionary changes in gene regulation of deeply conserved genes as being of primary importance in the regulation of very basic sociality. These results are in general agreement with predictions of the social ladder hypothesis, but further data on an even wider spectrum of social species within the carpenter bees can elucidate whether regulation of conserved genes gives way to protein sequence change and novel genes in later stages of sociality (Shell and Rehan 2018). 

## Supplementary Material

Supplementary Data
