# Supplementary material for: Conserved Genes Underlie Phenotypic Plasticity in an Incipiently Social Bee
Source: Genome Biol Evol. 2018 Sep 22;10(10):2749–58. doi: 10.1093/gbe/evy212 (PMC6190964; doi:10.1093/gbe/evy212)
Supplement: Supplementary Data [file evy212_supp.zip › GBE AustralensisSupporting Information-Sept7.docx]

**Conserved genes underlie phenotypic plasticity in an incipiently social bee**

Supplementary Methods

**Sample preparation and sequencing**

Two haploid *C. australensis* males were collected from Warwick, Queensland, Australia in December 2014, and DNA extracted using a Qiagen Genomic-tip 20/G kit (Valencia, CA) following standard protocol. Genomic DNA was then used to generate PCR-free 150bp DNA libraries for Illumina sequencing, performed at Genome Quebec. To improve genome assembly, genomic DNA was also extracted from the whole body of 15 female *C. australensis* as above, pooled, and used to construct 3kb, 5kb and 10kb mate pair libraries. Five paired end (PE) libraries with insert sizes ranging from 150bp to 10kb were constructed and sequenced on Illumina HiSeq 2500.

Before filtering this produced 219 Gb of raw sequence data (366 million read pairs, 2x150bp). Low quality reads, reads with a high proportion of Ns or poly-A structures were discarded and PE reads whose mates overlapped were merged prior to assembly. All genomic data have been submitted to public repositories and can be found using the NCBI BioProject number PRJNA302037.

Reads were checked for quality, using FastQC, both before and after correction/adapter removal (Patel and Jain 2012). Trimmomatic was then used to remove adapter contamination using Illumina Nextera-related adapter sequences (Bolger et al. 2014). Afterwards, FastUniq was used to remove duplicates from each library (Xu et al. 2012) and the SOAPdenovo’s error correction module (Luo et al. 2012) was then used to correct errors in the reads using a kmer size of 31. Post-filtering, this left 32 Gb of sequences (107,384,903 read pairs; Table S1).

REAPR was used to break putatively misassembled scaffolds at weak points (Hunt et al. 2013). We then used SSpace to reassemble broken scaffolds/contigs (Boetzer et al. 2011). Assembly gaps left after scaffolding were filled using Gapfiller (Boetzer and Pirvano 2012). Lastly, L_RNA_Scaffolder (Xue et al. 2013) was used along with independently assembled transcripts compiled by Trinity (Haas et al. 2013) to further scaffold contigs/scaffolds that were broken in gene annotations, followed by a final round of gap filling with GapFiller (Boetzer and Pirvano 2012). Completeness of the assembly was first assessed using the BUSCO pipeline (Simao et al. 2015) and the genome size was estimated using kmer-based error correction in KmerGenie (Chikhi and Medvedev 2014).

**Genome annotation**

The MAKER2 pipeline was used to perform gene annotations (Holt and Yandell 2011). Gene prediction algorithms of SNAP (Korf 2004), Augustus (Stanke and Waack 2003), and GeneMark (Lukashin and Borodovsky 1998) were iteratively trained for use in *C. australensis*. Annotations with a MAKER AED score above 0.75 or no significant InterProScan domains were flagged as weakly supported (Holt and Yandell 2011). Using default parameters, we identified repetitive elements using RepeatMasker (Smit et al. 2015) based on the Repbase transposable element (TE) library (‘species=all’) and a custom species-specific repeat library generated from RepeatModeler (Jurka et al. 2005). Genes were functionally annotated using Blast2GO (Conesa et al. 2005), with default settings for GO assignments, based on a querying of the NR database (01/05/2015) with BLASTP (E-value <10^-5^), along with all significant InterProScan hits. Using the KAAS server, KEGG orthology was assigned (Moriya et al. 2007).

Completeness of the genome assembly and gene annotation completeness were both assessed using Benchmarking Universal Single-Copy Orthologs (BUSCOs) (Simao et al. 2015). Specifically, the OrthoDB BUSCO pipeline (v.1.2; Oct. 2013) was employed to test for the presence as well as completeness of orthologs in relation to the *A. mellifera* sequences from arthropod-level BUSCOs in *C. australensis*.

**Gene family expansion**

Using default parameters in OrthoMCL (Li et al. 2003), orthologous groups were identified for analysis of gene family expansion, from *C. australensis*, as well as 11 other bee species: *Apis florea, A. mellifera, Bombus impatiens, B. terrestris, C. calcarata, Dufourea novaeangliae, Eufriesea mexicana, Habropoda laboriosa, Lasioglossum albipes, Melipona quadrifasciata* and *Megachile rotundata* into gene families. Only the longest transcript isoform for each gene was used, as well as genes with a minimum of 50 amino acid proteins. To identify gene families that have undergone significant size changes, we used the program COUNT (Csűros 2010). Count uses phylogenetic birth-and-death models in probabilistic inference to calculate the probability of gene family evolution across the phylogeny. As an input phylogenetic tree (Fig. S1), we used: ((((Ccal:55,Caust:55):50,(Hlab:92,((Mqua:68,(Bimp:13,Bter:13)

:55):10,(Emex:62,(Amel:19,Aflo:19):43):16):14):13):15,Mrot:106):9,(Dnov:85,Lalb:85):30) based on (Rehan et al. 2015). The rate optimization was computed by COUNT using the default parameters and posterior probabilities for each gene family were given. Using an in-house R script, these gene families were then described using Pfam and Interpro (Finn et al. 2014) domains and keywords and subsequently, sorted into gene ontology terms (Ashburner et al. 2000).

**Taxonomically restricted gene analysis**

We performed a BLASTX search using longest-isoform protein-coding *C. australensis* nucleotide sequences against a protein RefSeq database (accessed 02/15/17). Significance was determined with an e-value of < 10^-5^ and the ETE toolkit was utilized to obtain the common ancestral taxonomic level of the significant BLAST hits for each gene with a local NCBI database (Huerta-Cepas et al. 2016). We thus obtained raw phylostratigraphic levels (“phylostrata”) for each gene, and each raw level was condensed into one of eight phylostrata: Cellular, Eukaryota, Bilateria, Insecta, Hymenoptera, Apoidea, Apidae, and *Ceratina*. Genes placed in intermediate levels were condensed to the phylostratum below, thus ensuring that each gene was at least as old as its assigned level (Table S13). Thus, relative minimum age for each protein-coding gene was estimated. Statistical significance assessed using Pearson’s chi-square test between DEGs and non-DEGS for six different categories; overall DEGs, genes upregulated in solitaries, social primaries, social secondaries, reproductives (upregulated in solitary and social primary females), and non-reproductives (social secondaries and pre-dispersal females), as well as old (cellular-Insecta) and new (Hymenoptera*-Ceratina*) phylostratigraphic levels. Additionally, chi-square tests of independence were performed at each phylostrata level within each of the phylostrata levels as well.

**Protein evolution and selection**

For alignments used in PAML analyses we identified sequences with homology to putative 1-to-1 ortholog groups previously established among 11 bee genomes by OrthoDB v.8 (Kriventseva et al. 2015). To identify genes subject to positive selection (dN/dS >1) and/or with high rates of protein evolution in *C. australensis*, we first mapped 3978 single-copy protein-coding orthologous sequences of *C. australensis* to 11 other bee species from OrthoDB v.8. Sequence data came from the following sources: *C. calcarata* from (Rehan et al. 2015); *A. mellifera* from Consortium datasets OGSv3.2 (Elsik et al. 2014); and the rest from the 10 bee project (Kapheim et al. 2015), which includes *L. albipes* OGSv5.42 provided by (Kocher et al. 2013). These orthologous amino acid sequences were then aligned using PRANK (with the +F parameter) in the Guidance v2.01 wrapper (Sela et al. 2015; Löytynoja and Goldman 2005), producing multiple sequence alignments and initial gene trees for each orthologous group. Gene trees were then improved with 100 iterations of RAxML v.8.2.9 using the PROTGTRGAMMA model (Stamatakis 2014). Amino acid alignments were converted to in-frame nucleotide alignments with Pal2Nal v.14 (Suyama et al. 2006). Of the 3978 orthologous groups, 445 groups contained inconsistencies between protein and nucleotide sequences, and were incompatible with the Pal2Nal program.

Rates of synonymous substitutions (dS) and nonsynonymous substitutions (dN) and the nonsynonymous to synonymous ratio (dN/dS) were calculated for each orthologous group with the codeml program in PAML v.4.8 (Yang 2007), using the free-ratio model (model = 1; NSites = 0). For comparisons of evolutionary rate parameters to gene expression data we filtered out genes whose alignment length (used by PAML) was < 80 and those with estimated dN/dS was greater than 6 – the later filter was applied because all genes with a dN/dS > 6 lacked any synonymous changes along the *C. australensis* branch, and were thus unreliable.

For evaluating significant differences in evolutionary rates we performed branch tests on all ortholog groups as follows: Two models were used for hypothesis testing: the null model estimated a single dN/dS value for all sequences in the orthologous group (model = 0; NSsites = 0); while the alternative model used a branch test (model = 2; NSsites = 0) specifying individual species as foreground branches and the rest as background branches. We performed two tests in this way: Test 1 had *C. australensis* as the only member of the foreground branch, while Test 2 had both *C. australensis* and *C. calcarata* as foreground branch species to represent subfamily Xylocopinae. Thus, we could use these tests to determine if either *C. australensis* or Xylocopinae had a significantly different rate of evolution relative to background species. The log-likelihoods of each model were then used to calculate a likelihood ratio test statistic. Significance was determined by comparing these statistics to a Chi-squared distribution with one degree of freedom, with a value of 0.05 as significance threshold. P-values were then corrected for multiple comparisons using the Benjamini-Hochberg method (Benjamini and Hochberg 1995).

We also utilized PAML to perform branch site tests, using the branch-site A model. For this test, we used the same approach as for the branch Test 1 noted above, comparing our null (H0) model which assumes a fixed dN/dS value along the foreground branch (model = 2; NSsites = 2; fix_omega = 1; omega = 1), to our alternative (H1) model which allows dN/dS to independent along the foreground branch (model = 2; NSsites = 2; fix_omega = 0; omega = 1). We then only considered genes to be significant if the adjusted p-value (Benjamini and Hochberg 1995) was below 0.05 and at least one site was significant (>95%) using the Bayes empirical Bayes (BEB) test.

**Gene expression and GO enrichment**

The R (Version 3.2.4) package DESeq (Anders and Huber 2010) from the Bioconductor repository was used to test for differential expression among pairwise behavioral types, between foraging and reproductive categories (social primaries and solitary females vs. social secondaries and pre-dispersal females), between different age classes (pre-dispersal females vs. solitary active brood and social behavioral categories), as well as between social primaries vs. all other behavioral categories. Additionally, the likelihood ratio test in DESeq2 (Love et al. 2014) (Version 1.16.1), as well as the generalized linear model in edgeR (Robinson et al. 2010) were used to test for significant differentially expressed genes. These methods showed largely similar expression patterns (Supplemental Table S17 & S18, with the DESeq results being the most conservative and used for subsequent analyses (DESeq=1591, DESeq2=2674, edgeR=1862 DEGs).

Heatmaps of scaled read counts were constructed using the R package heatmap.2 in gplot (Version 2.12.1) (Warnes et al. 2012). Statistical support for hierarchical clustering topologies within the heatmap was constructed using bootstrap resampling probabilities, implemented in the R package pvclust (Suzuki and Shimodaira 2006). Additionally, pairwise hypergeometric tests were performed on DEG lists from previously stated comparisons to determine whether there was statistically significant GO enriched terms using Blast2GO (FDR ≤ 0.05; two-tailed).

**Prediction of *cis*-regulatory elements**

C*is*-regulatory binding motifs associated with differentially expressed genes were identified using AME in the MEME program suite (Bailey et al. 2009; McLeay and Bailey 2010). Windows 5kb upstream of all differentially expressed genes (N=1591) were used in this analysis and checked for motifs from fly factor survey and JASPAR core insect database (Zhu et al. 2011; Portales-Casamar et al. 2010) (p < 0.001 after Bonferroni correction). Upstream regions of 5318 non-differentially expressed genes were also used as control sequences in AME.

**Comparative transcriptomics**

Differentially expressed genes (DEGs), significantly enriched GO terms, and overrepresented transcription factor binding motifs were compared by reciprocal best blast hits to data from 44 studies related to aggression, dominance, subordinance, queen and worker behavior, as well as development, across 27 different taxa (Table S16). Homologous sequences of DEGs between *C. australensis* and other species were identified using tBLASTx (E-value ≤ 1e-4). Significant overlap to these studies was performed using a two-tailed hypergeometric test.

REFERENCES

Ashburner M, et al (2000) Gene ontology: tool for the unification of biology. *Nat Genet* 25:25-29.

Berens AJ, Hunt JH, Toth AL (2014) Comparative transcriptomic of convergent evolution: different genes but conserved pathways underlie caste phenotypes across lineages of eusocial insects. *Mol Ecol Res* 32:690-703.

Berens AJ, Hunt JH, Toth AL (2015) Nourishment level affects castes-related gene expression in *Polistes* wasps. BMC Genomics. 16:235.

Benjamini Y, Hochberg Y (1995) Controlling the false discovery rate: a practical and powerful approach to multiple testing. *J R Stat Soc Series B* 57:289-300.

Blatti C, Sinha S (2014) Motif Enrichment Tool. *Nucleic Acids Res* 42:W20-W25.

Bolger AM, Lohse M, Usadel B (2014) Trimmomatic: a flexible trimmer for Illumina sequence data. *Bioinformatics* 30:2114-2120.

Boetzer M, Henkel CV, Jansen HJ, Butler D, Pirovano W (2011) Scaffolding pre-assembled contigs using SSPACE. *Bioinformatics* 27:578-579.

Boetzer M, Pirovano W (2012) Toward almost closed genomes with GapFiller. *Genome Biol* 13: R56.

Bonasio R. et al. (2010) *Science* 329:1068-1071.

Buitenhuis B, Hedegaard J, Janss L, Sorensen P (2009) Differentially expressed genes for aggressive pecking behavior in laying hens. *BMC Genomics* 10:544.

Chen X, et al. (2012) A Transcriptome comparison between honey bee queen and worker destined larvae. *Insect Biochem Mol Biol*. 42:665-673.

Chikhi R, Medvedev P (2014) Informed and automated k-mer size selection for genome assembly. *Bioinformatics* 30:31-37.

Colgan TJ, et al. (2011) Polyphenism in social insects: insights from a transcriptome-wide analysis of gene expression in the life stages of the key pollinator, *Bombus terrestris*. *BMC Genomics* 12:623.

Csűros M (2010) Count: evolutionary analysis of phylogenetic profiles with parsimony and likelihood. *Bioinformatics* 26:1910-1912.

Elsik CG, Worley KC, Bennett AK, Beye M, Camara F, Childers CP, de Graaf DC, Debyser G, Daugherty TH, Toth AL, Robinson GE (2011) Nutrition and division of labor: effects of foraging and brain gene expression in the paper wasp *Polistes metricus*. *Mol Ecol* 20:5337-5347.

Deng J, Devreese B, Elhaik E, Evans JD, Foster LJ, Graur D, Guigo R, *et al.* (2014) *BMC Genomics* 15:86.

Finn RD, et al. (2014) The Pfam protein families database. *Nucleic Acids Res* 42:222-230.

Graff J, Jemielity S, Parker JD, Parker KM, Keller L (2007) Differential gene expression between adult queens and workers in the ant *Lasius niger*. *Mol Ecol* 16:675–683.

Grant CE, Bailey TL, Noble WS (2011) FIMO: Scanning for occurrences of a given motif. *Bioinformatics* 27:1017–1018.

Greenwood AK, Peichel CL (2015) Social regulation of gene expression in three spine sticklebacks. *PLOS ONE* 10:e0137726.

Grozinger CM, Sharabash NM, Whitfield CW, Robinson GE (2003) Pheromone-mediated gene expression in the honey bee brain. *Proc Natl Acad Sci USA* 100:14519–14525.

Haas BJ, et al. (2013) *De novo* transcript sequence reconstruction from RNA-seq: reference generation and analysis with Trinity. *Nat Protoc* 8:1494–1512.

Hollis F, et al. (2015) Mitochondrial function in the brain links anxiety with social subordination. *Proc Natl Acad Sci USA* 112:15486-15491.

Huerta-Cepas, Jaime, François Serra, and Peer Bork (2016) ETE 3: Reconstruction, analysis, and visualization of phylogenomic data. *Mol Biol Evol* 33.6:1635-1638.

Hunt JH, et al. (2010) Differential gene expression and protein abundance evince ontogenetic bias toward castes in a primitively eusocial wasp. *PLOS ONE* 5:e10674.

Hunt M, et al. (2013) REAPR: a universal tool for genome assembly evaluation. *Genome Biol* 14: R47.

Holt C, Yandell M (2011) MAKER2: an annotation pipeline and genome-database management tool for second-generation genome projects. *BMC Bioinformati*c 12:491.

Jurka J, Kapitonoc VV, Pavlicek A, Klonowski P, Walichiewicz J (2005) Repbase update, a database of eukaryotic repetitive elements. *Cytogenet Genome Res* 110:462–467.

Kocher SD, Ayroles JF, Stone, EA, Grozinger CM (2010) Natural variation in pheromone response correlates with reproductive traits and brain gene expression in worker honey bees. *PLOS ONE* 5:e9116.

Kocher, SD, Li, C, Yang, W, Tan, H, Yi, SV, Yang, X, Hoekstra, HE, Zhang, G, Pierce, NE, Yu, DW. (2013). The genome of a socially polymorphic halictid bee, *Lasioglossum albipes*. *Genome Biol* 14(12):R142.

Korf I (2004) Gene finding in novel genomes. *BMC Bioinformatics* 5:59.

Kriventseva EV, et al. (2015) OrthoDB v8: update of the hierarchical catalog of orthologs and the underlying free software. *Nucleic Acids Res* 43:D250–256.

Liang ZS, et al. (2014) Comparative brain transcriptomic analyses of scouting across distinct behavioral and ecological contexts in honeybees. *Proc R Soc Lond B* 281:20141868.

Lopes JS, Abril-de-Abreu R, Oliveira RF (2015) Brain transcriptomic response to social eavesdropping in zebrafish (*Danio rerio*). *PLOS ONE* 10:e0145801.

Love MI, Huber W, Anders S (2014) Moderated estimation of fold change and dispersion for RNA-seq data with DESeq2. Genome Biol 15:550. doi: 10.1186/S15059-014-0550-8.

Löytynoja A, Goldman N (2005) An algorithm for progressive multiple alignment of sequences with insertions. *Proc Natl Acad Sci USA* 102:10557-10562.

Lukashin AV, Borodovsky M (1998) GeneMark.hmm: new solutions for gene finding. *Nucleic Acids Res*26:1107–1115.

Luo R, et al. (2012) SOAPdenovo2: an empirically improved memory-efficient short-read de novo assembler. *GigaScience* 1:18.

Manfredini F, et al. (2013) Sociogenomics of cooperation and conflict during colony founding in the fire ant *Solenopsis invicta*. *PLOS Genet* 9:e1003633.

Manfredini F, et al. (2014) Molecular and social regulation of worker division of labour in fire ants. *Mol Ecol* 23:660-672.

Manfredini F, Brown MJF, Vergoz V, Oldroyd BP (2015) RNA-sequencing elucidates the regulation of behavioural transitions associated with the mating process in honey bee queens. *BMC Genomics* 16:563.

Matys V, et al. (2003) TRANSFAC: transcriptional regulation, from patterns to profiles. *Nucleic Acids Res* 31:374–378.

McLeay R, Bailey TL (2010) Motif Enrichment Analysis: A unified framework and method evaluation. *BMC Bioinformatics* 11:165.

Moriya Y, Itoh M, Okuda S, Yoshizawa AC, Kanehisa M (2007) KAAS: an automatic genome annotation and pathway reconstruction server. *Nucleic Acids Res* 35:182–185.

Oliveira RF, et al. (2016) Assessment of fight outcome is needed to activate socially driven transcriptional changes in the zebra-fish brain. *Proc Natl Acad Sci USA* 113:654–661.

Patel RK, Jain M (2012) NGS QC Toolkit: a toolkit for quality control of next generation sequencing data. *PLOS ONE* 7:e30619.

Portales-Casamar E, et al. (2010) JASPAR 2010: the greatly expanded open-access database of transcription factor binding profiles. *Nucleic Acids Res* 38: D105–D110.

Renn SCP, Aubin-Horth N, Hofmann HA (2008) Fish and chips: function genomics of social plasticity in an African cichlid fish. *J Exp Biol* 211:3041-3056.

Robinson MD, McCarthy DJ, Smyth GK (2010) edgeR: a Bioconductor package for differential expression analysis of digital gene expression data. *Bioinformatics* 26:1.

Romiguier J, Cameron SA, Woodard SH, Fischman BJ, Keller L, Praz CJ. 2016. Phylogenomics controlling for base compositional bias reveals a single origin of eusociality in corbiculate bees Molec Biol Evol 33:670–678.

Sanogo YO, Band M, Blatti C, Sinha S, Bell AM (2012) Transcriptional regulation of brain gene expression in response to a territorial intrusion. *Proc R Soc Lond [Biol]* 279:4929-4938.

Sarma MS, Whitfield CW, Robinson GE (2007) Species differences in brain gene expression profiles associated with adult behavioral maturation in honey bees. *BMC Genomics* 8:202.

Sela I, Ashkenazy H, Katoh K, Pupko T (2015) GUIDANCE2: accurate detection of unreliable alignment regions accounting for the uncertainty of multiple parameters. *Nucleic Acids Res* 43:W7–W14.

Simao FA, Waterhouse RM, Ioannidis P, Kriventseva EV, Zdobnov EM (2015) BUSCO: assessing genome assembly and annotation completeness with single-copy orthologs. *Bioinformatics* doi: 10.1093/bioinformatics/btv351.

Smit AFA, Hubley R, Green P (2015) RepeatMasker Open-4.0. <http://www.repeatmasker.org>.

Sumner S, Pereboom JJ, Jordan WC (2006) Differential gene expression and phenotypic plasticity in behavioral castes of the primitively eusocial wasp, *Polistes canadensis*. *Proc R Soc Lond B* 273:19-26.

Stamatakis A (2014) RAxML version 8: a tool for phylogenetic analysis and post-analysis of large phylogenies. *Bioinformatics* 30:1312–1313.

Suyama M, Torrents D, Bork P (2006) Pal2Nal: robust conversion of protein sequence alignments into the corresponding codon alignments. *Nucleic Acids Res* 34:W609-W612.

Stanke M, Waack S (2003) Gene prediction with a hidden Markov model and a new intron submodel. *Bioinformatics* 19:215-225.

Suzuki R, Shimodaira H (2006) Pvclust: an R package for assessing the uncertainty in hierarchical clustering. *Bioinformatics* 22:1540-1542.

Thompson GJ, Kucharski R, Maleszka R, Oldroyd BP (2006) Towards a molecular definition of worker sterility: differential gene expression and reproductive plasticity in honey bee. *Insect Mol Biol* 15:537-644.

Toth AL, et al. (2007) Wasp gene expression supports an evolutionary link between maternal behaviour and eusociality. *Science* 318:441-444.

Vojvodic S, et al. (2015) The transcriptomic and evolutionary signature of social interactions regulating honey bee caste development. *Ecol Evol* 5:4795-4807.

Warnes GR, et al. (2012) gplots: various R programming tools for plotting data. R package version 2.12.1. <http://CRAN.R-project.org/package=gplots>.

Woodard SH, Bloch GM, Band MR, Robinson GE (2014) Molecular heterochrony and the evolution of sociality in bumblebees (*Bombus terrestris*). *Proc R Soc Lond B* 281:20132419.

Xu H, et al. (2012) FastUniq: A fast *de novo* duplicates removal tool for paired short reads. PLOS ONE 7:e52249.

Yang Z (2007) PAML 4: Phylogenetic Analysis by Maximum Likelihood. *Mol Biol Evol* 24:1586-1591.

Zhu LJ, et al. (2011) FlyFactorSurvey: a database of *Drosophila* transcription factor binding specificities determined using the bacterial one-hybrid system. *Nucleic Acids Res* 39:D111–D117.

FIGURES


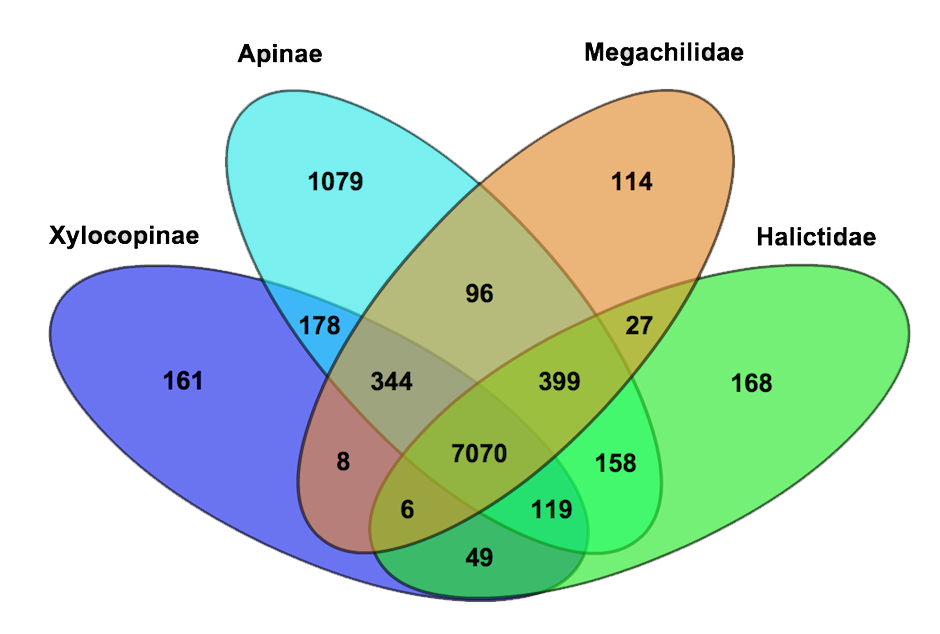


**Figure S1**. Gene family overlap among four bee lineages 1) subfamily Xylocopinae (*C. australensis* and *C. calcarata*), versus 2) subfamily Apinae (*A. florae, A. mellifera, B. impatiens, B. terrestris, E. mexicana, H. laboriosa* and *M. quadrifasciata*), versus 3) family Megachilidae (*Megachile rotundata*), and versus 4) (*L. albipes* and *D. novaeangliae*). Numbers indicate the gene families in each comparison.

**Figure S2.** Phylogeny of bees with sequenced genomes, including *C. australensis,* depicting divergence dates estimated by (Kapheim et al. 2015, Rehan et al. 2016, Romiguier

et al. 2016). Divergence dates shown for each node in millions of years before present (MYA).

**Figure S3.** Distribution of upregulated genes from reproductive categories (social primary and solitary females) and non-reproductive categories (social secondaries and pre-dispersal females) across phylostratigraphic levels. Asterisks indicate chi-square test of independence significance between DEGS and non-DEGS for each phylostrata (p < 0.05). See Table S14 for chi square statistics.

**Figure S4.** Distribution of upregulated genes from social primaries and social secondaries across phylostratigraphic levels**.** Asterisks indicate chi-square test of independence significance between DEGS and non-DEGS for each phylostrata (p < 0.05). See Table S14 for chi square statistics.

**Figure S5.** Distribution of upregulated genes from social primaries and solitary females (X^2^=42.14, df=7, p < 0.01) across phylostratigraphic levels. Asterisks indicate chi-square test of independence significance between DEGS and non-DEGS for each phylostrata (p < 0.05). See Table S14 for chi square statistics.
